# Transcriptome Analysis Revealed Ameliorative Effects of *Bacillus* Based Probiotic on Immunity, Gut Barrier System, and Metabolism of Chicken under an Experimentally Induced *Eimeria tenella* Infection

**DOI:** 10.3390/genes12040536

**Published:** 2021-04-07

**Authors:** Fareed Uddin Memon, Yunqiao Yang, Imdad Hussain Leghari, Feifei Lv, Ahmed M. Soliman, Weiyu Zhang, Hongbin Si

**Affiliations:** 1 College of Animal Sciences and Technology, Guangxi University, Nanning 530004, China; fareedjanm@gmail.com (F.U.M.); yyqop01@163.com (Y.Y.); 1918393033@st.gxu.edu.cn (F.L.); ahmed.mahmoud8933@yahoo.com (A.M.S.); zweiyu@gxu.edu.cn (W.Z.); 2 Faculty of Animal Husbandry and Veterinary Sciences, Sindh Agriculture University, Tando Jam 70060, Pakistan; ihleghari@sau.edu.pk; 3 Agricultural Research Center, Biotechnology Department, Animal Health Research Institute, Giza 12618, Egypt

**Keywords:** *Bacillus subtilis*, *Eimeria tenella*, chicken, transcriptome analysis, immunity, metabolism

## Abstract

In this study, we performed transcriptome analysis in the cecum tissues of negative control untreated non-challenged (NC), positive control untreated challenged (PC), and *Bacillus subtilis* (*B. subtilis*) fed challenged chickens (BS + ET) in order to examine the underlying potential therapeutic mechanisms of *Bacillus* based probiotic feeding under an experimental *Eimeria tenella* (*E. tenella*) infection. Our results for clinical parameters showed that birds in probiotic diet decreased the bloody diarrhea scores, oocyst shedding, and lesion scores compared to positive control birds. RNA-sequencing (RNA-seq) analysis revealed that in total, 2509 up-regulated and 2465 down-regulated differentially expressed genes (DEGs) were detected in the PC group versus NC group comparison. In the comparison of BS + ET group versus PC group, a total of 784 up-regulated and 493 down-regulated DEGs were found. Among them, several DEGs encoding proteins involved in immunity, gut barrier integrity, homeostasis, and metabolism were up-regulated by the treatment of probiotic. Functional analysis of DEGs also revealed that some gene ontology (GO) terms related with immunity, metabolism and cellular development were significantly affected by the exposure of probiotic. Kyoto encyclopedia of genes and genomes (KEGG) pathway analysis showed that the DEGs in the cecum of *B. subtilis*-fed challenged group were mainly participated in the pathways related with immunity and gut barrier integrity, included mitogen-activated protein kinase (MAPK) signaling pathway, toll-like receptor (TLR) signaling pathway, extracellular matrix (ECM)–receptor interaction, tight junction, and so on. Taken together, these results suggest that *Bacillus* based probiotic modulate the immunity, maintain gut homeostasis as well as barrier system and improve chicken metabolism during *E. tenella* infection.

## 1. Introduction

Coccidiosis, one of the most common parasitic disease in chicken caused by seven different intestinal habitual species of the genus *Eimeria*, including *E. tenella*, *E. maxima*, *E. necatrix*, *E. mitis*, *E. brunetti*, *E. praecox,* and *E. acervulina* [1]. *E. tenella* has been found one of the most common and pathogenic species, which occupies in the ceca of chicken and causes sever destruction of cecal epithelial cells and necrosis of the gut barrier, resulting in reduced nutrient absorption, decreased weight gain, and productivity of bird [2,3]. Currently in poultry industry, coccidiosis is mainly controlled by coccidiostats and live vaccines. However, prolonged use of anti-coccidial agents has emerged drug resistance and elevated the public health concerns of food safety regarding drug residues in food and food products. On the other hand, live vaccines are highly expensive to produce, and their use can cause pathogenic strain reversion [4]. In this regard, potential alternatives and safe approaches should be emerged to treat the *Eimeria* infection.

In recent decades, bioactive compounds including probiotics, prebiotics, phytobiotics, antimicrobial peptides, essential oils, and enzyme supplements have been used as alternative therapies to treat various intestinal diseases [5,6]. Among them, probiotics have found to be effective in improving the growth rate, enhancing the activity of gut beneficial bacteria, improving the integrity of gastrointestinal tract, stimulating the innate and acquired immunity and maintaining the gut homeostasis during intestinal infectious diseases [7,8]. *B. subtilis* is a common probiotic and widely used in poultry industry because of its ability to produce spore, which can tolerate the extreme environments such as low and high temperatures, different levels of pH, bile and enzymes encountered in upper gastro-intestinal tract [9]. Previous studies have demonstrated that *Bacillus* based probiotics could enhance the functional activities of immune organs, regulate Th1-Th2 balance, enhance barrier functions, stimulate signaling pathways related with immune activator, thus initiating the specific and nonspecific immunity of the host, and developing the performance of animals [10,11].

Analysis of RNA-sequencing (RNA-seq) is an efficient technique to examine the pathological effects of disease and screening the mechanisms of drugs at tissue and/or cell level by investigating the whole transcriptional responses of host to such pathogen and drug [12]. Several studies have examined the mechanisms of probiotics on transcriptional levels by low-throughput techniques, such as quantitative polymerase chain reaction (qPCR) and/or northern blotting [13,14]. However, the use of these methods is limited and can be explored to measure single gene expression levels [15]. Therefore, the present study was carried out for the first time to examine the efficacy of probiotic to counteract the negative effects of *Eimeria* on chicken gut, a pan genomic analysis of the transcriptomic fingerprint was conducted in the ceca of control chickens, chickens challenged with *E. tenella* in the presence and absence of probiotic treatment.

## 2. Materials and Methods

### 2.1. E. tenella and Probiotic

The strain of *E. tenella* was isolated from the infected ceca of chicken in the field in Guangxi, China, and the oocysts were harvested, sporulated, and stored in the Department of Clinical Veterinary Medicine, Collage of Animal Sciences and Technology, Guangxi University, Nanning, China, as previously described in our study [16].

*Bacillus* based probiotic was purchased from Kangjialong Feed co., LTD China (201906157), contained *B. subtilis* at the concentration of 1 × 10^8^ cfu/g.

### 2.2. Ethical Statement

All experimental conditions for animals were performed according to the guidelines approved by the Animal Care and Use Committee of Guangxi University, Nanning, China. The specific ethical approval code is Gxu-2019-180.

### 2.3. Experimental Design

A total of 63 one-day old Chinese native yellow chickens were purchased from a hatchery and reared in 9 coccidia-free cages with free access to feed and water for up to 28 days. Upon arrival, all chicks were weighed and randomly divided into one of three experimental groups with 21 birds in each group. The three groups consisted of: (1) negative control group (NC), chickens received normal diet; (2) positive control group (PC), chickens received normal diet; (3) *B. subtilis*-fed group (BS + ET), chickens received diet supplemented with *B. subtilis* probiotic throughout the experimental trial (day 1 to day 28 of age) at the dose of 1g/kg of feed. On day 21 of age, the chickens in group PC and BS + ET chickens were challenged with 6 × 10^4^ *E. tenella* sporulated oocysts, whereas the chickens in group NC were gavaged with normal saline to provide same management stress. Challenged and non-challenged groups were housed in two different rooms for avoiding the transmission of disease. Temperature (32 to 34 °C for first week and then decreased by 2 °C per week), humidity (60 to 80%) and light (23 h L:1D) of both rooms were maintained during the entire period of experiment.

### 2.4. Evolution of Clinical Parameters and Sample Collection for RNA-Sequencing 

On day 5, 6, and 7 post-infection (PI), fecal droppings were examined for bloody diarrhea scores (0–4) according to the technique described by Morehouse and Baron [17]. At the same time, fecal samples were collected and collected samples were used to count the oocysts on McMaster chamber slide as previously described by Hodgson [18].

On day 7 post-infection, 3 individual chickens per group were slaughtered by direct heart puncture and their ceca were scored (0–4) for lesions as described by Johnson and Reid [19]. At the same time, approximately 2 cm long cecal tissue samples of all groups were collected for analyzing the whole transcriptional responses to the exposure of probiotic under an induced *E. tenella* infection in chickens.

### 2.5. RNA Extraction

For the extraction of total RNA from all collected samples, a modified TRIzol method [20] was used and the RNA was extracted according to the instructions of TRIzol Kit manual (B518651-0100, Sangon Biotech co, Ltd., Shanghai, China). The purity of RNA was determined using NanoPhotometer^®^ Spectrophotometer (IMPLEN, CA, USA) and the concentration of total RNA was determined with Qubit^®^ 2.0 Fluorimeter by using Qubit^®^ RNA Assay Kit (Life Technologies, South San Francisco, CA, USA). RNA purity and integrity were further measured with Bioanalyzer 2100 system using RNA Nano Assay Kit (Agilent Technologies, Santa Clara, CA, USA). The RNA samples those passed the concentration, purity, and integrity tests without any degradation and contamination were used for transcriptome sequencing.

### 2.6. Library Construction for Illumina Sequencing

A total amount of 1.5 µg RNA per sample was used as input material for the RNA sample preparation. NEBNext^®^ Ultra™ Library Preparation Kit for Illumina^®^ (NEB, Ispawich, CA, USA) was used to generate the sequencing libraries in accordance with the manufacture’s recommendation and index code of each sample was added to attribute sequences. Finally, PCR products were purified (AMPure XP system) and the quality of generated libraries was measured with Bioanalyzer 2100 system.

### 2.7. Clustering and Sequencing

cBOT Cluster Generation System was used to carried out the clustering of index-coded samples by using HiSseq 4000 PE Cluster Kit (Illumina) following manufacture’s manual. After generating the clusters, the paired-end sequencing was performed on Illumia Hiseq 4000 Platform and 150 paired-end reads were generated.

### 2.8. Raw Data Processing and Analysis

Raw data were recorded in a FASTQ format through in-house Perl scripts. In this step, low-quality data such as reads with a linker, poly N and low quality reads were removed to obtain clean data. In addition, the quality parameters of Q20, GC content and repetitive sequence level were measured in order to ensure the accuracy of data analysis. The reference genome was downloaded from the genome website (ftp://ftp.ensembl.org/pub/release102/fasta/gallus_gallus/DNA/Gallus_gallus.GRCg6a.DNA.toplevel.fa.gz). Bowtie v2.0.6 software was used to construct the reference genome index [21] and TopHat v2.0.9 software [22] was used to compare the paired-end clean reads to the reference genome. The HTSeq software was used to count the number of reads mapped to the genome of each sample [23].

### 2.9. Screening of Differentially Expressed Genes

After homogenizing the expression levels of each differential gene by reads per kilo bases per million reads (RPKM), the differentially expressed genes of all experimental groups were screened with DESeq2 software [24] and the standard for the screening was: log 2 fold change ≥ 0.58 and corrected *p*-value < 0.05. The correction of *p*-value was performed according to the method established by Benjamini and Hochberg [25].

### 2.10. Gene Ontology and Kyoto Encyclopedia of Genes and Genomes Enrichment Analysis of Differentially Expressed Genes

The functional enrichment analysis was implemented using GOseq R package [26]. In this step, all significant differentially expressed genes were enriched into GO terms. The analysis of KEGG pathways was carried out using KOBAS (2.0) software [27], in which the DEGs were compared with the KEGG database and all pathways those participated with differentially expressed genes were determined.

### 2.11. Validation of DEGs by qRT-PCR

For the validation of RNA-seq data, 8 DEGs were selected from the comparison of BS + ET versus PC groups. Primers used for the validation are described in Table S1. *β-actin* gene was used as internal reference gene. Quantitative real time PCR (qRT-PCR) for selected DEGs was performed using 2×SG fast qPCR Master mix SYBR-green (B639271-0005, Sangon Biotech co, Ltd., Shanghai, China). The consistency of RNA-seq results was tested and each sample was repeated for 3 times. The transcriptional levels of each gene relative to reference gene were calculated by 2^−ΔΔCt^ method [28].

### 2.12. Statistical Analysis

Collected data of bloody diarrhea, oocyst shedding, and lesion scores were subjected to an analysis of variance (ANOVA) using mixed procedure of SPSS version 20.0 (SPSS Inc., Chicago, IL, USA) and their results in table were presented as least square means with standard error of mean (*n* = 3/group). The results of selected DEGs for validation were subjected to grouped table of Graphpad Prism 5.0 software (Graphpad, San Diego, CA, USA) and analyzed by two-way ANOVA. Significant differences were compared using Tukey test and the statistical difference was accepted at *p* < 0.05.

## 3. Results

### 3.1. Clinical Parameters

The results of clinical parameters are shown in Table 1. No bloody diarrhea scores, oocyst shedding in feces, and cecal lesions were detected in negative control group. The significant low bloody diarrhea scores (4–7 PI), oocyst shedding in feces (4–7 PI) and cecal lesions were observed in *E. tenella* infected group that was fed diet supplemented with *Bacillus* based probiotic compared with that in non-supplemented challenged group.

### 3.2. Data Analysis of Transcriptome

To explore the potential molecular mechanisms of *Bacillus* based probiotic in preventing the *Eimeria* infection in chickens, we performed RNA sequencing analysis on the cecal tissue samples in three groups (control negative, *E. tenella* challenged untreated and *E. tenella* challenged treated with probiotic) respectively. As shown in Table 2, RNA sequencing of the nine cecal libraries (three replicate samples/group) generated 19,842,850 to 20,963,369 150 bp paired-end raw reads per library, while the GC content was reached between 46.94% and 51.46%. These findings indicate high abundance of RNA sequencing and relative consistency of GC content for each sample. Following trimming and filtering the reads with adapters, low quality reads and the ratio of poly N greater than 10%, we obtained 18,807,009 to 20,110,347 clean reads that had a length of 52.4 gigabases (Gb). The total comparison rate was about 91.06% and over 86.77% reads aligned uniquely to the reference genome. These findings further confirm the reliability of transcriptome analysis and the accuracy of cecal tissue samples used in the present study.

### 3.3. Differentially Expressed Genes Regulated by the Exposure of E. tenella and Probiotic

**Figure 1:**
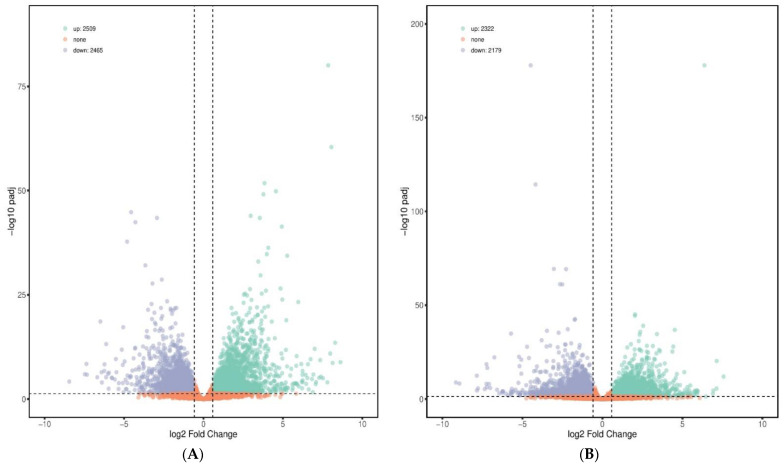

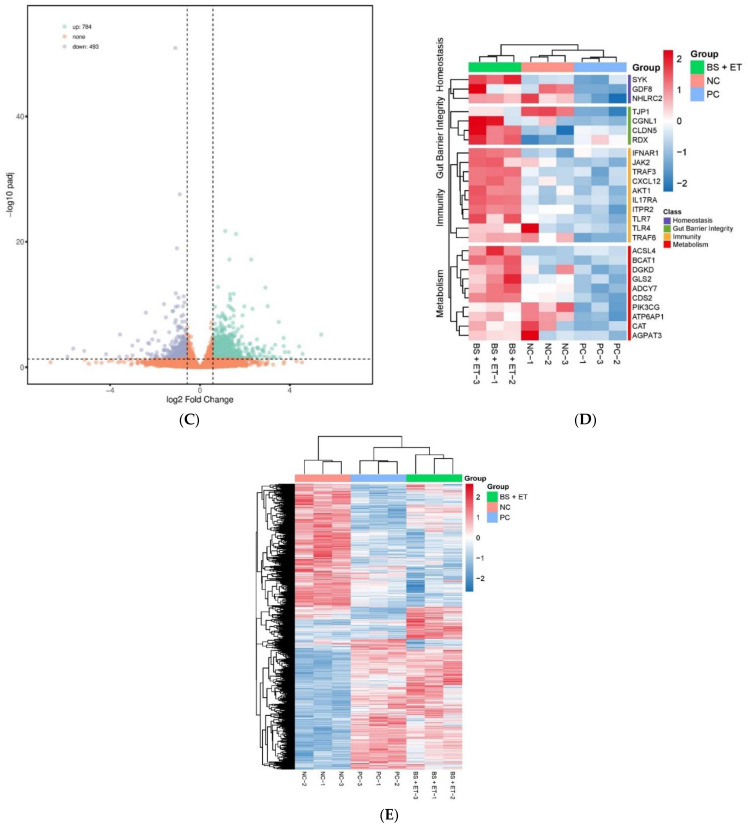
Results of differentially expressed genes (DEGs) affected by treatments. (**A**) The number of DEGs (up-regulated and down-regulated) in the PC group versus NC group comparison. (**B**) The number of DEGs (up-regulated and down-regulated) in the BS + ET group versus NC group comparison. (**C**) The number of DEGs (up-regulated and down-regulated) in the BS + ET group versus PC group comparison. **(D)** Results of DEGs related with metabolism, immunity, homeostasis, and gut barrier integrity. **(E)** Cluster analysis of DEGs between NC, PC, and BS + ET groups.

In total, 2509 up-regulated and 2465 down-regulated DEGs were detected in the PC group versus NC group; 2322 up-regulated and 2179 down-regulated DEGs were found in the BS + ET versus NC groups, whereas 784 up-regulated and 493 down-regulated DEGs were found in BS + ET group versus PC group (Figure 1A–C and Table S2).

Several genes encoding proteins involved in immunity were up-regulated in response to probiotic exposure when compared with *E. tenella* challenged untreated group. They mainly include *TNF receptor-associated factor 3* (*TRAF3*), *TNF receptor-associated factor 6* (*TRAF6*), *C-X-C motif chemokine 12* (*CXCL12*), *toll-like receptor 4* (*TLR4*), *toll-like receptor 7* (*TLR7*), *interferon α and β receptor subunit 1* (*IFNAR1*), *AKT serine/threonine kinase 1* (*AKT1*), *inositol 1,4,5-triaphosphate receptor type 2* (*ITPR2*), *interleukin 17 receptor A* (*IL17RA),* and *janus kinase 2* (*JAK2*). Some genes that maintain gut barrier integrity were also up-regulated with log 2 fold change 0.58: *tight junction protein 1* (*TJP1*), *radixin* (*RDX*), *claudin 5* (*CLDN5*), and *cingulin like 1* (*CGNL1*). Genes associated with cellular and tissue homeostasis (*MSTN*, also known as *GDF8*, *NHLRC2,* and *SYK*) were showed up-regulated transcriptional levels in group BS + ET when compared with PC group. Furthermore, genes involved in metabolism (*ACSL4*, *ADCY7*, *AGPAT3*, *ATP6AP1*, *BCAT1*, *CAT*, *CDS2*, *DGKD*, *GLS2*, *PIK3CG,* and so on) were also showed up-regulated transcriptional levels in infected group fed probiotic diet compared to that in infected group fed normal diet (Figure 1D and Table S2).

### 3.4. Gene Ontology Enrichment Analysis for Unique Differentially Expressed Genes

**Figure 2:**
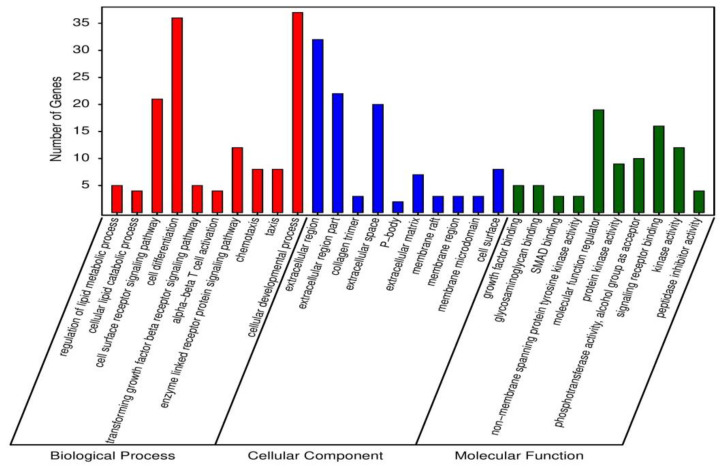
Top 30 GO terms enriched of up-regulated DEGs in BS + ET group versus PC group.

In order to examine the mechanism of probiotic supplementation at genetic level, 1277 differentially expressed unique genes were processed for GO enrichment analysis between PC and BS + ET groups. A total of 118, 4 and 10 GO terms of up-regulated DEGs, while 136, 24 and 25 GO terms of down-regulated DEGs were significantly enriched in biological process, cellular component, and molecular function (Table S3). Top 30 enriched GO terms of up-regulated DEGs are presented in Figure 2. GO terms that were enriched in biological process, included regulation of lipid metabolic process, cellular lipid catabolic process, cell surface receptor signaling pathway, cell differentiation, transforming growth factor β receptor signaling pathway, α-β T cell activation, enzyme linked receptor protein signaling pathway, chemotaxis, taxis, and cellular developmental process.

GO terms associated with cellular components were extracellular region, extracellular region part, collagen trimer, extracellular space, P-body, extracellular matrix, membrane raft, membrane region, membrane microdomain and cell surface.

Top ten GO terms that were enriched in the category of molecular function, including growth factor binding, glycosaminoglycan binding, SMAD binding, non-membrane spanning protein tyrosine kinase activity, molecular function regulator, protein kinase activity, phosphotransferase activity, alcohol group as acceptor, signaling receptor binding, kinase activity and peptidase inhibitor activity.

### 3.5. Analysis of KEGG Pathways

**Figure 3:**
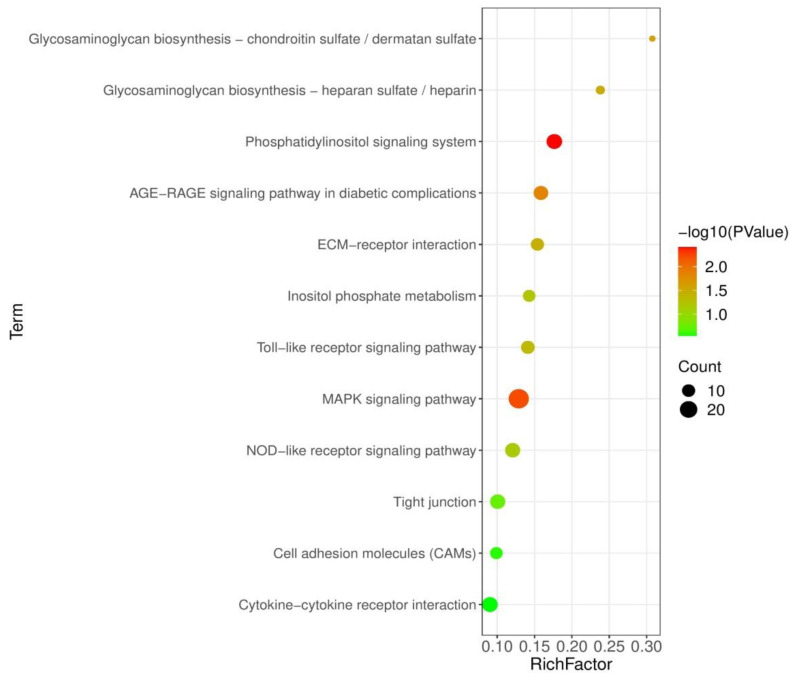
KEGG pathways significantly modulated in BS + ET group versus PC group comparison.

To examine the pathways affected by probiotic feeding, 1277 DEGs were then mapped to the KEGG database for pathways enrichment analysis. Figure 3 shows the KEGG pathways that were enriched in BS + ET group compared to PC group. Phosphatidylinositol signaling system, AGE-RAGE signaling pathway in diabetic complications, MAPK signaling pathway were the most significantly enriched of up-regulated DEGs. Other pathways such as toll-like receptor signaling pathway, nucleotide-binding oligomerization domain-like (NOD-like) receptor signaling pathway, cytokine–cytokine receptor interaction, extracellular matrix (ECM) receptor interaction, cell adhesion molecules (CAMs) and tight junction were also significantly enriched (Table S4).

GO terms and KEGG pathways enrichment analysis between NC and PC groups were also carried out and their results are shown in Tables S3 and S4.

### 3.6. Validation of DEGs by qRT-PCR

**Figure 4:**
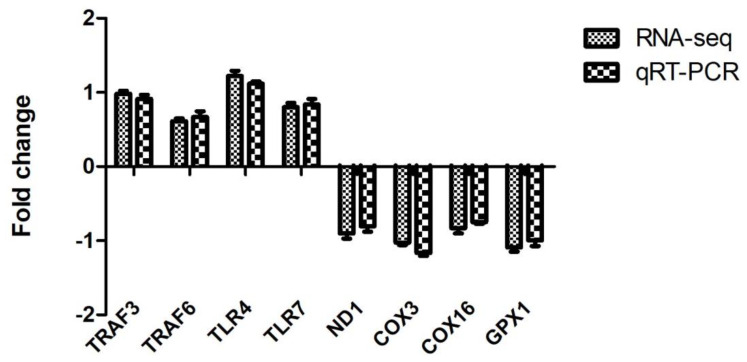
Validation of RNA-seq by qRT-PCR.

In order to verify the reliability of RNA-seq data, 4 up-regulated (*TRAF3*, *TRAF6*, *TLR4*, and *TLR7*) and four down-regulated DEGs (*ND1*, *COX3*, *COX16,* and *GPX1*) were selected for qRT-PCR. Our results revealed that the obtained fold change values by qRT-PCR were highly consistent with the values of RNA-seq (Figure 4). The consistency between qRT-PCR and RNA-seq indicate the reliability of transcriptome analysis.

## 4. Discussion

Bloody diarrhea scores, oocyst counting in feces, and lesion scoring are considered the main clinical observations for examining the severity of *Eimeria* infection. In the present study, increased bloody feces, oocyst shedding in feces, and cecal lesions were observed in *E. tenella* challenged untreated birds. These findings indicated that *Eimeria* infection induced gut inflammation thereby increased the blood in feces, oocyst shedding and cecal lesions. However, for the *E. tenella* challenged birds, bloody diarrhea scores, oocyst shedding in feces, and cecal lesions were significantly decreased with *B. subtilis* feeding when compared with that in infected untreated birds. These results are in line with the findings of other studies that reported that supplementation of probiotic reduced bloody feces, oocyst excretion, and lesions during coccidia infection [29,30]. These beneficial effects imply that the probiotic feeding reduces the gut inflammation caused by *Eimeria*.

It is well known that *Eimeria* infection alters nutrient digestion and absorption, perturbs physiological, and metabolic homeostasis in gut and depresses immune system of host [31,32]. Several studies have shown that the probiotic microbes improve nutrient digestibility, maintain gut homeostasis by balancing gut microbiota, improve gut barrier integrity, defend against various intestinal pathogen invasion and adhesion, and modulate humoral and cellular immunity [33,34]. Based on these modes of actions of probiotics microbes, transcriptome analysis was performed to examine the beneficial effects of probiotic in response to the deleterious effects of *Eimeria* infection. Our results for DEGs revealed that several genes encoding proteins involved in immunity, gut barrier integrity, homeostasis, and metabolism were up-regulated in the cecum samples of the infected birds that received probiotic diet when compared with infected birds fed normal diet. These results are in great agreement with the previous findings that showed that probiotic feeding modulated DEGs associated with immunity, homeostasis, and metabolism when the whole transcriptome analysis was carried out in the gut tissue samples of chickens [35], piglets [36], and fishes [37]. Beneficial microbes are well known to modulate immune and metabolic related genes as well as pathways that positively influence the physiological status and overall performance of the host [38]. Furthermore, early exposure of probiotic microbes plays an essential role in developing the immune components that have long lasting effects on the physiology of host [39].

To understand the functions of DEGs and their interaction with the biological pathways, GO and KEGG analysis were performed. Our results demonstrated that various GO terms and KEGG pathways related with immunity, barrier integrity, and metabolism were significantly enriched in the probiotic supplemented group when compared with control positive untreated group. The major enriched KEGG pathways were MAPK signaling pathway, toll-like receptor signaling pathway, NOD-like receptor signaling pathway, cytokine–cytokine receptor interaction, cell adhesion molecules, ECM–receptor interaction, tight junction, and some metabolic pathways (Figure 3 and Table S4).

**Figure 5:**
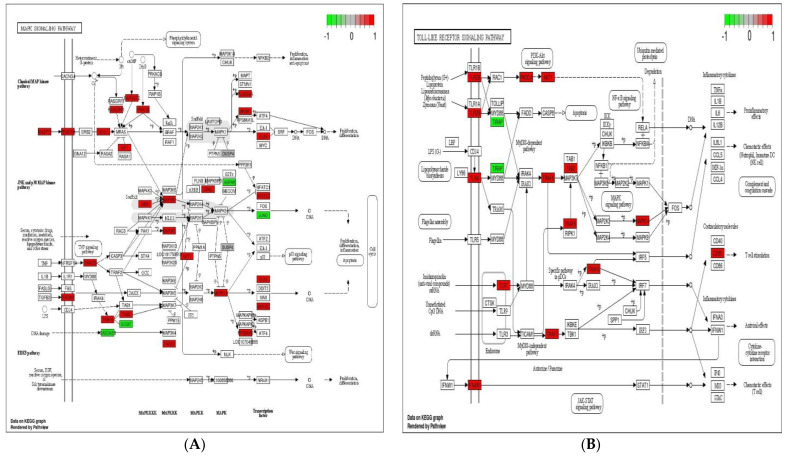

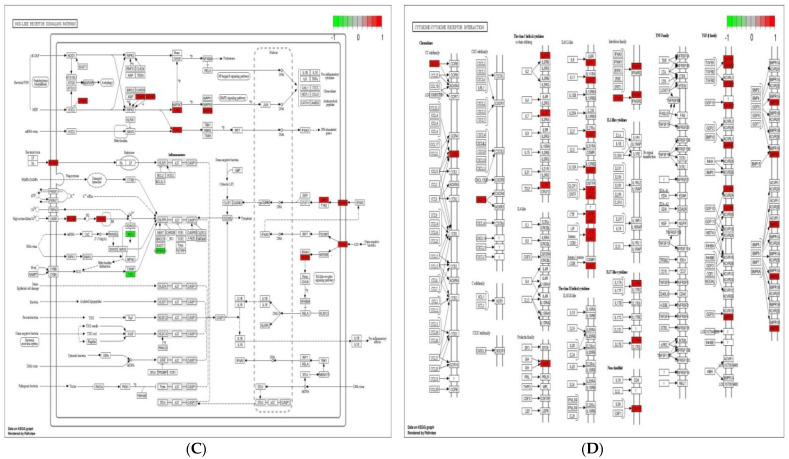
Significantly modulated KEGG pathways in BS + ET group versus PC group comparison. (**A**) Expression patterns of the DEGs of MAPK signaling pathway. (**B**) Expression patterns of the DEGs of toll-like receptor signaling pathway. (**C**) Expression patterns of the DEGs of NOD-like receptor signaling pathway. (**D**) Expression patterns of the DEGs of cytokine–cytokine receptor interaction. Highlighted genes with red color represent up-regulated expression patterns and highlighted genes with green color represent down-regulated expression patterns.

Immune system plays a key role in protecting the host from invading pathogens. During the course of infection, multiple signaling pathways are activated in immune cells, which confer defense mechanism against pathogens. Toll-like receptor, NOD-like receptor (NLR) and MAPK signaling pathways are considered the 1st line defense mechanism against pathogens and function in the regulation, proliferation, and survival of immune cells [40,41]. The stimulation of TLR and NLR in response to pathogen invasion leads to activate innate immune cells such as macrophages and neutrophils, repair damaged tissues, and trigger other signaling pathways such as MAPK signaling pathway, which control several cellular activities in immune system and regulate inflammatory cytokines [41]. The initiation of these cytokines then triggers the activities of chemokines and/or adhesion molecules at the sites of infection to clear the pathogens [42]. In the present experiment, 10, 11, 23, and 15 up-regulated DEGs were enriched in toll-like receptor signaling pathway, NOD-like receptor signaling pathway, MAPK signaling pathway, and cytokine–cytokine receptor interaction in response to probiotic treatment when compared with non-treated challenged group (Figure 5). These findings suggest that the supplementation of *B. subtilis* possibly triggered the toll-like receptor signaling pathway and NOD-like receptor signaling pathway, which resulting activation of MAPK signaling pathway and cytokines to confer the defense mechanism against *Eimeria*.

**Figure 6:**
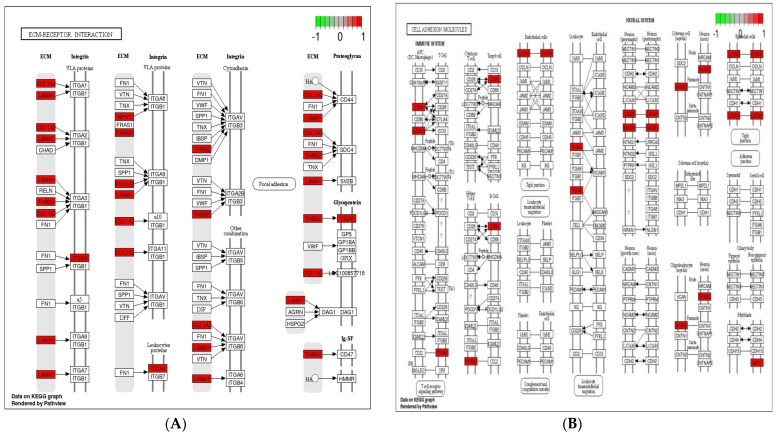

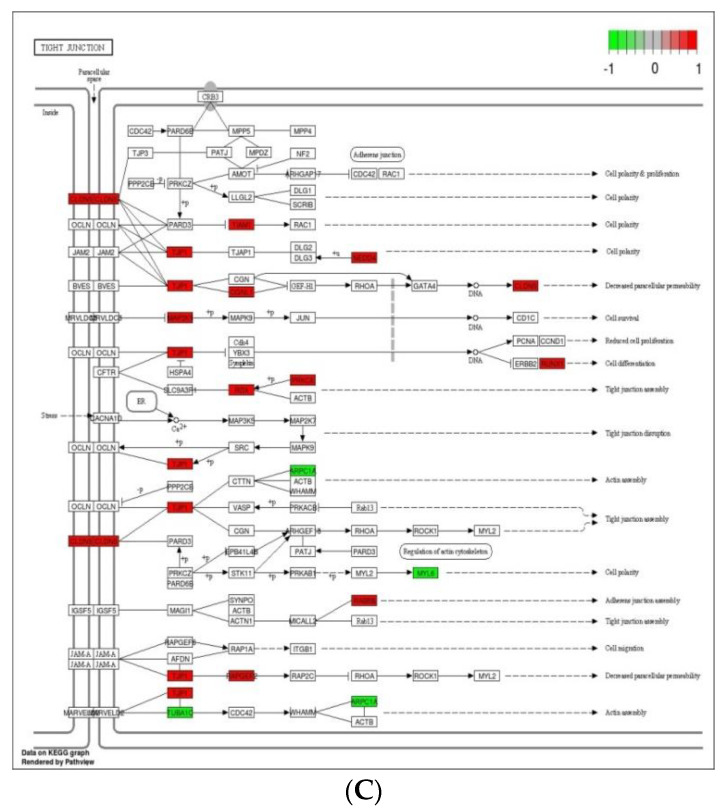
Significantly modulated KEGG pathways in BS + ET group versus PC group comparison. (**A**) Expression patterns of the DEGs of ECM–receptor interaction. (**B**) Expression patterns of the DEGs of cell adhesion molecules. (**C**) Expression patterns of the DEGs of tight junction. Highlighted genes with red color represent up-regulated expression patterns and highlighted genes with green color represent down-regulated expression patterns.

Cell adhesion molecules are protein structures located on the surfaces of cells, which function in binding the cell with other cell or cell with ECM [43]. ECM comprises several structural and functional macromolecules that function in morphogenesis of tissues and organs and maintain the function and structure of cells and tissues. Interactions between cells and ECM are initiated by cell surface associated components or transmembrane molecules, which control various cellular activities such as adhesion, proliferation, migration, and differentiation [44]. The cell to cell and/or cell to ECM interaction is mainly takes place at the site of tight junctions (TJs) that block the paracellular pathways between epithelial cells and form physical barriers against microbial infections. However, a large number of pathogens target the cell adhesion molecules in order to invade epithelial cells and disrupt epithelial integrity to access the deeper tissues for dissemination. Therefore, maintaining these processes is essential to sustain the cellular activities and epithelial integrity [45]. The enriched DEGs in cell adhesion molecules, ECM–receptor interaction, and tight junction (Figure 6) herein imply that *B. subtilis* has ability to maintain the structures and functions of cells and epithelial integrity regardless of *Eimeria* challenge.

## 5. Conclusions

In summary, positive effects on bloody diarrhea scores, oocyst shedding, and lesion scores due to probiotic feeding exhibit potential therapeutic mechanisms. Transcriptome analysis revealed that several DEGs and GO terms related with immunity, homeostasis, barrier system, and metabolism were affected by the exposure of probiotic. Moreover, supplementation of *B. subtilis* activated MAPK signaling pathway, toll-like receptor signaling pathway, NOD-like receptor signaling pathway, cytokine–cytokine receptor interaction, cell adhesion molecules, ECM–receptor interaction, and tight junction, which improve the immunity and maintain gut barrier integrity following *Eimeria* infection. These findings provide evidence that *B. subtilis* can ameliorate *Eimeria* infection and can be used as an alternative to anti-coccidial drug.

## Figures and Tables

**Table 1:** Effects of *B. subtilis* on bloody diarrhea scores, oocyst shedding, and lesion scores.

Parameters		Groups	
	NC	PC	BS + ET
**Lesion scores (Grade)**			
Day 7 post-infection	0.00 ± 0.00 ^c^	3.00 ± 0.00 ^a^	1.66 ± 0.33 ^b^
**Bloody diarrhea scores**			
Day 5 post-infection	0.00 ± 0.00 ^c^	2.33 ± 0.33 ^a^	1.33 ± 0.33 ^b^
Day 6 post-infection	0.00 ± 0.00 ^c^	3.33 ± 0.33 ^a^	1.66 ± 0.33 ^b^
Day 7 post-infection	0.00 ± 0.00 ^c^	3.00 ± 0.57 ^a^	1.33 ± 0.33 ^b^
**Oocyst counting (OPG/g × 10^3^)**			
Day 5 post-infection	0.00 ± 0.00 ^c^	34.00 ± 2.51 ^a^	20.33 ± 1.20 ^b^
Day 6 post-infection	0.00 ± 0.00 ^c^	51.33 ± 4.05 ^a^	28.66 ± 2.18 ^b^
Day 7 post-infection	0.00 ± 0.00 ^c^	77.66 ± 2.90 ^a^	37.33 ± 3.17 ^b^

Superscript letters (^a–c^) show significant difference. Results are presented as mean ± standard error of mean (*n* = 3). NC = Negative control untreated non-challenged group; PC = positive control untreated challenged group; BS + ET = *B. subtilis*-fed challenged group.

**Table 2:** Summary of RNA-sequencing results and quality data output.

Sample	Raw Reads	Raw Bases (G)	Clean Reads	Clean Bases (G)	Q20 (%)	GC Content (%)
NC-1	20,395,539	6.12	19,319,443	5.80	97.28	46.94
NC-2	19,948,996	5.98	18,807,009	5.64	97.73	47.16
NC-3	20,615,240	6.18	19,233,599	5.77	97.49	48.18
PC-1	20,963,369	6.29	19,772,595	5.93	96.96	51.25
PC-2	19,983,927	6.00	19,174,592	5.75	96.94	50.95
PC-3	20,852,755	6.26	20,110,347	6.03	96.86	51.46
BS + ET-1	20,851,951	6.26	20,048,311	6.01	96.79	51.06
BS + ET-2	19,842,850	5.95	18,763,598	5.63	97	51.19
BS + ET-3	20,570,711	6.17	19,463,192	5.84	96.92	50.16
Sum	184,025,338	55.51	174,692,686	52.4		

## Data Availability

The metadata, processed data and raw data associated with this study have been deposited in GEO NCBI under the accession number GSE166905 that can be accessed for review with the secure token: qxqpgacqvrepjkp.

## Supplementary Materials

The following tables are available online at https://www.mdpi.com/article/10.3390/genes12040536/s1, Table S1: Primers used for the validation of RNA-seq by qRT-PCR. Table S2: DEGs affected by treatments, Table S3: GO terms affected by treatments: Table S4: KEGG pathways affected by treatments.
