# Supplementary material for: Transcriptome Analysis Revealed Ameliorative Effects of Bacillus Based Probiotic on Immunity, Gut Barrier System, and Metabolism of Chicken under an Experimentally Induced Eimeria tenella Infection
Source: Genes (Basel). 2021 Apr 7;12(4):536. doi: 10.3390/genes12040536 (PMC8067821; doi:10.3390/genes12040536)
Supplement: Supplementary file 1 [file genes-12-00536-s001.zip › Supplementary Table 1.docx]

**Table 1:** Primers used for the validation of RNA-seq by qRT-PCR

| **Genes** |  | **Primer Sequence (5_3_)** | **Gene Bank ID** |
| --- | --- | --- | --- |
| *β-actin* | F | GAGAAATTGTGCGTGACATCA | L08165 |
|  | R | CCTGAACCTCTCATTGCCA |  |
| *TRAF3* | F | CCAGCTCTCAGCAGCAGGAGACA | BX935958 |
|  | R | TCAGCACGAGGACACGGAAGC |  |
| *TRAF6* | F | ATGGAAGCCAAGCCAGAGTT | XM015287208 |
|  | R | ACAGCGCACCAGAAGGGTAT |  |
| *TLR4* | F | AGTCTGAAATTGCTGAGCTCAAAT | NM001030693 |
|  | R | GCGACGTTAAGCCATGGAAG |  |
| *TLR7* | F | CCTGACCCTGACTATTAACCAT | NM001011688 |
|  | R | CGTAAAGTAGCAGGAAGACCC |  |
| *ND1* | F | ACCATTCCCCCTTGCAGACC | AB753758.1 |
|  | R | GGCTCGAAGGGCTCCGATTA |  |
| *COX3* | F | TCAAGCCTAGCCCCAACACC | KC847975.1 |
|  | R | TGCTGTGGTGAGCCCATGTA |  |
| *GPX1* | F | AACCAATTCGGGCACCAG | HM590226 |
|  | R | CCGTTCACCTCGCACTTCTC |  |
| *COX16* | F | GGAGCGGTTCCGCAAACTG | [NM_001197057.1](https://www.ncbi.nlm.nih.gov/entrez/viewer.fcgi?db=nucleotide&id=308080013) |
|  | R | TGAGCAAACTCACGGAGTCCA |  |
